# Spin/valley pumping of resident electrons in WSe_2_ and WS_2_ monolayers

**DOI:** 10.1038/s41467-021-25747-5

**Published:** 2021-09-15

**Authors:** Cedric Robert, Sangjun Park, Fabian Cadiz, Laurent Lombez, Lei Ren, Hans Tornatzky, Alistair Rowe, Daniel Paget, Fausto Sirotti, Min Yang, Dinh Van Tuan, Takashi Taniguchi, Bernhard Urbaszek, Kenji Watanabe, Thierry Amand, Hanan Dery, Xavier Marie

**Affiliations:** 1 Université de Toulouse, INSA-CNRS-UPS, LPCNO, 135 Av. Rangueil, 31077 Toulouse, France; 2 Physique de la matière condensée, Ecole Polytechnique, CNRS, IP Paris, 91128 Paris, Palaiseau France; 3 Department of Electrical and Computer Engineering, University of Rochester, Rochester, NY 14627 USA; 4 International Center for Materials Nanoarchitectonics, National Institute for Materials Science, 1-1 Namiki, Tsukuba, 305-00044 Japan; 5 Research Center for Functional Materials, National Institute for Materials Science, 1-1 Namiki, Tsukuba, 305-00044 Japan; 6 Department of Physics, University of Rochester, Rochester, NY 14627 USA

**Keywords:** Two-dimensional materials, Two-dimensional materials

## Abstract

Monolayers of transition metal dichalcogenides are ideal materials to control both spin and valley degrees of freedom either electrically or optically. Nevertheless, optical excitation mostly generates excitons species with inherently short lifetime and spin/valley relaxation time. Here we demonstrate a very efficient spin/valley optical pumping of resident electrons in n-doped WSe_2_ and WS_2_ monolayers. We observe that, using a continuous wave laser and appropriate doping and excitation densities, negative trion doublet lines exhibit circular polarization of opposite sign and the photoluminescence intensity of the triplet trion is more than four times larger with circular excitation than with linear excitation. We interpret our results as a consequence of a large dynamic polarization of resident electrons using circular light.

## Abstract (web summary)

Optical excitation of transition metal dichalcogenide monolayers mostly generates excitons species with inherently short lifetime and spin/valley relaxation time. Here, the authors demonstrate efficient spin/valley optical pumping of resident electrons in n-doped WSe_2_ and WS_2_ monolayers.

## Introduction

Transition metal dichalcogenides (TMD) such as MoS_2_, MoSe_2_, WS_2_, or WSe_2_ are layered semiconductors with promising applications in optoelectronics and spintronics^1^. In the monolayer (ML) limit, they become direct band gap semiconductors, with gaps located at the six corners of the hexagonal Brillouin zone (K valleys)^2–4^. Remarkably, they exhibit a strong light-matter interaction governed by tightly bound excitons with binding energies of several hundreds of meV^5^. In addition, they are characterized by a strong spin-orbit coupling and a lack of crystal inversion symmetry resulting in original spin/valley properties^6–9^. Among them, chiral optical selection rules dictate that circularly polarized light can photo-generate carriers in either K or K′ valleys with either spin up or spin down, i.e. the so-called spin/valley pumping. Thus, TMD MLs were quickly considered as an ideal platform to control both spin and valley degrees of freedom with potential applications in quantum information processing^10–13^. Nevertheless, light excitation usually yields neutral excitons and using these photo-generated species to encode spin or valley information is inherently limited by both their short recombination time (~ps)^14–16^ and their very fast spin/valley relaxation time induced by electron-hole exchange interaction (~ps)^17,18^. Recently other strategies have been proposed using longer lived excitonic species such as dark excitons, dark trions, or interlayer excitons in heterostructures^19–21^. Another promising route consists in using resident electrons or holes in doped monolayers. Beyond its obvious advantage for future devices as compared to the manipulation of excitons, the spin/valley relaxation of resident carriers is prevented by spin/valley locking and is not governed by efficient exchange interaction like for excitons. Spin/valley relaxation times as long as 100’s ns to several µs for electrons and holes have been measured in WSe_2_ using time-resolved Kerr experiments and spin/valley noise spectroscopy^22–25^. Nevertheless, very little is known about the polarization mechanism and the maximum degree of polarization one can reach for resident carriers. Back et al.^26^ showed that a near complete valley polarization of electrons can be reached in a n-doped MoSe_2_ ML but it requires an out-of-plane magnetic field of 7 T that is incompatible with the development of future devices.

In this letter, we demonstrate a very efficient spin/valley pumping mechanism which yields very large polarization for resident electrons in n-doped WSe_2_ and WS_2_ monolayers following a circularly polarized excitation without applying any magnetic field. In contrast to pump-probe experiments, we use continuous-wave (cw) laser excitation that leads to a dynamical building of this very large polarization. We use the degree of circular polarization of the photoluminescence associated with negative trions as probes of the polarization of electrons (both the intervalley triplet trion X^T−^ and the intravalley singlet trion X^S−^ which consist in the binding of a photo-generated electron-hole pair with a resident electron from the opposite (same) valley (see Fig. 3a below)). In n-WSe_2_ we measure a very large positive circular polarization 90% for the triplet trion and a negative polarization −40% for the singlet trion. Remarkably, the total intensity of the triplet trion following circular excitation is more than four times larger than the total intensity following linear excitation. Using simple models of trion formation, we demonstrate that all these observations are consistent with a very efficient spin/valley pumping of resident electrons and give an estimate of ~80% for polarization.

## Results

### Sample description

**Fig. 1:**
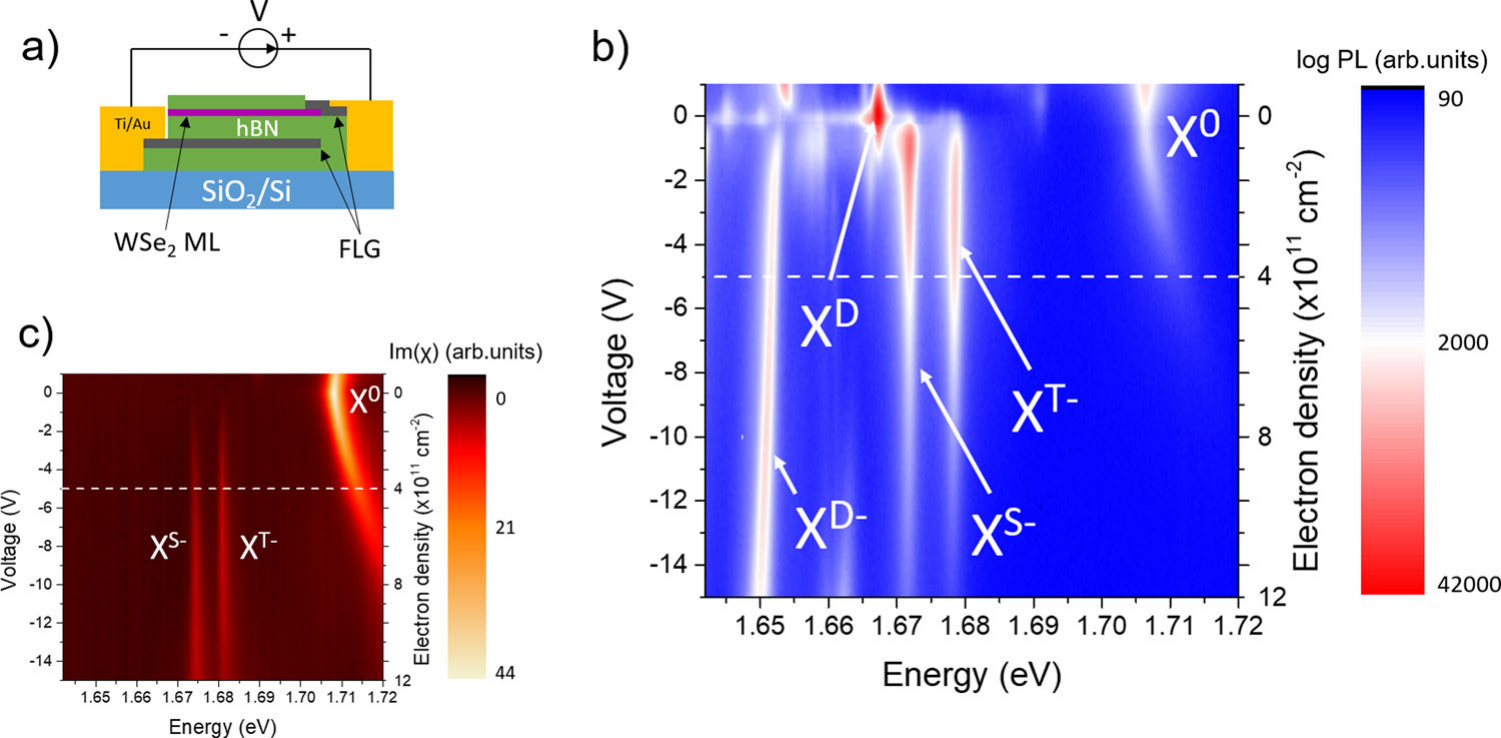
Excitonic states in n-doped WSe_2_. **a** Sketch of the sample. **b** PL intensity as a function of electron density. The excitation energy is 1.96 eV. **c** Imaginary part of the optical susceptibility χ measured using differential reflectivity as a function of electron density. The reflectivity contrast is transformed into Im(χ) using a Kramers-Kronig transform^26^.

We fabricate a high quality WSe_2_ charge tunable device as sketched in Fig. 1a. Details of the sample fabrication can be found in the methods section. By tuning the voltage bias between a back gate and the ML we can electrostatically dope the ML. The estimation of the carrier density is presented in the Supplementary Note 1. We then perform polarization dependent micro-photoluminescence (PL) experiments in the n-doping regime at a temperature of 4 K. The excitation source is the 632.8 nm line of a HeNe laser. Unless otherwise stated, the excitation power is 5 µW focused to a spot size smaller than 1 µm diameter. We also study a naturally n-doped WS_2_ ML without charge tuning with a cw 570 nm laser (excitation power of 18 µW and temperature of 20 K). More details on the experimental setup can be found in the methods section. Importantly, we restrict our study to moderate electron densities of a few 10^11^ cm^−2^ so that the simple three particles picture (i.e. trions) is equivalent to the many-body picture (i.e. Fermi polarons)^27,28^.

We first present in Fig. 1b the PL color plot as a function of bias in the charge tunable WSe_2_ device. We recognize several exciton species including the bright neutral exciton (X^0^), the dark neutral exciton (X^D^), the bright trions (intervalley triplet X^T−^ and intravalley singlet X^S−^), and the dark trion (X^D−^), in agreement with previous studies^29–37^. The spectral linewidth of X^0^ at the neutrality point is as low as 2.5 meV (FWHM) vouching for the state-of-the-art quality of the sample^34^. In Fig. 1c, we also present the color plot of the reflectivity contrast highlighting transitions with large oscillator strength (i.e. X^0^, X^T−^, and X^S−^). In the following we will focus on an electron doping density of 4 × 10^11^ cm^−2^ (see white dashed line in Fig. 1b, c) where X^T−^ and X^S−^ dominate the PL spectrum.

### Circular polarization

**Fig. 2:**
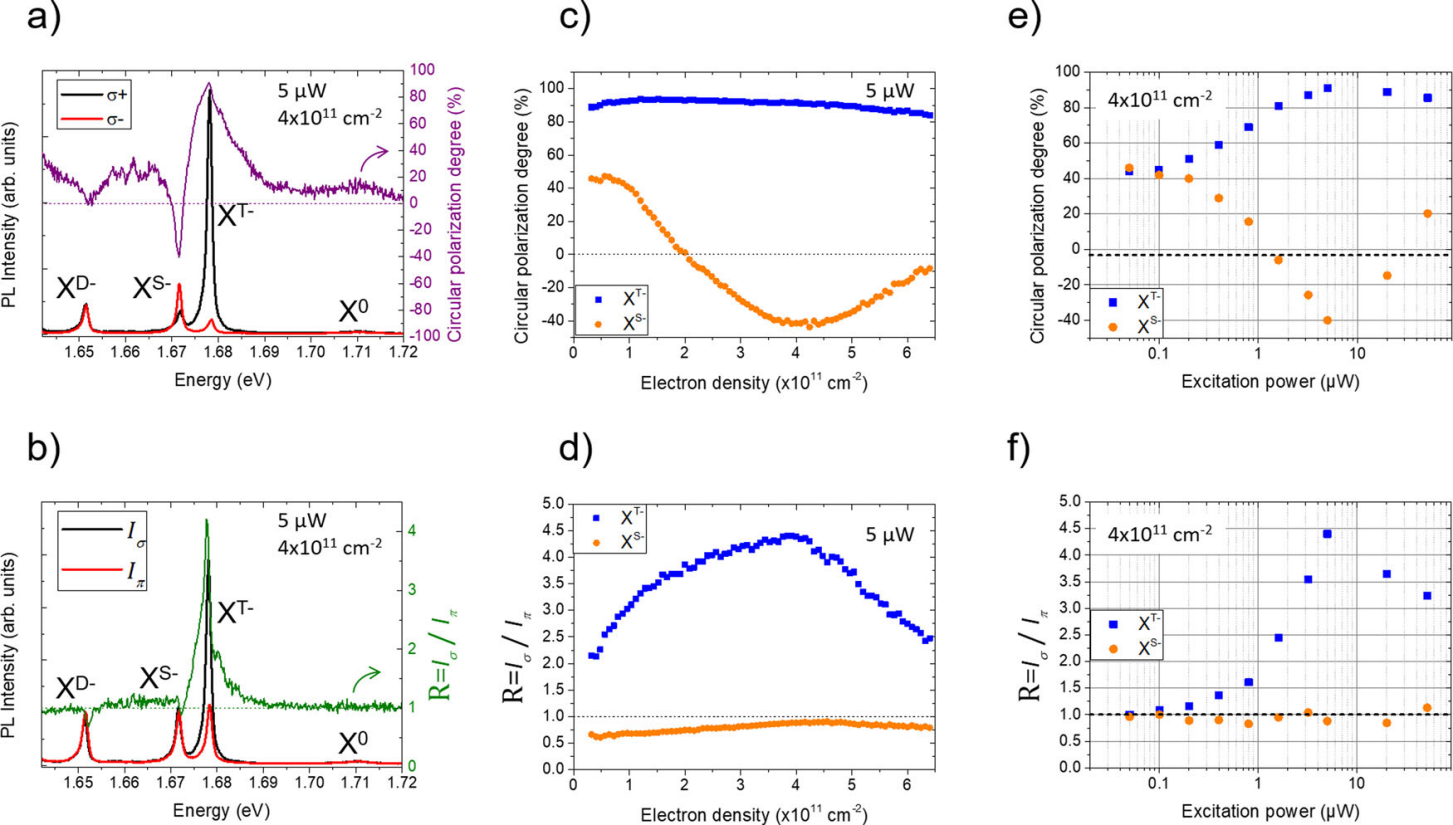
Experimental observations in n-WSe_2_. **a** Photoluminescence and circular polarization spectra for σ+ and σ− detections with σ+ excitation. **b** Total photoluminescence spectra with circular excitation and linear excitation. **c** Circular polarization degree at the peak of triplet and singlet negative trions as a function of electron density. **d** Ratio of PL intensities between circular and linear excitations at the peak for both triplet and singlet as a function of electron density. **e** Circular polarization degree at the peak of triplet and singlet as a function of excitation power. **f** Ratio of PL intensities between circular and linear excitations at the peak for both triplet and singlet as a function of excitation power.

Figure 2 presents the key results of this work. In Fig. 2a, we show the photoluminescence spectra for both σ+ and σ− detections following σ+ excitation. We define the degree of circular polarization as ${P}_{{{{{{\mathrm{c}}}}}}}=\frac{{I}_{\sigma +}-{I}_{\sigma -}}{{I}_{\sigma +}+{I}_{\sigma -}}$ where *I*_σ+_, *I*_σ−_ are the PL intensities with σ+ and σ- detection respectively. While the bright exciton X^0^ exhibits a positive circular polarization below +20% as a consequence of the efficient long-range exchange interaction, the lines of the bright trion doublet show strong polarization of opposite sign: +91% for X^T−^ and −40% for X^S−^ at the peak. Note that this negative polarization on the singlet has been observed elsewhere recently in state-of-the-art samples^34^. The dark trion X^D−^ shows no circular polarization in agreement with its out-of-plane polarization^31,36,37^.

### Circular and linear excitation

Then we switch to linear excitation π_*X*_ and measure both co-linear *I*_*X*_ and cross-linear *I*_*Y*_ intensities. We define the total PL intensity following linear excitation as *I*_*π*_ = *I*_*X*_ + *I*_*Y*_ and the total intensity following circular excitation as *I*_σ_ = *I*_σ+_ + *I*_σ−_ We show in Fig. 2b both *I*_*π*_ and *I*_*σ*_ and the ratio $R=\frac{{I}_{\sigma }}{{I}_{\pi }}$. There is no difference in intensity for X^0^ between linear and circular excitation (i.e. *R* = 1). On the other hand, *R* reaches a very large value of 4.4 at the peak of X^T−^ and slightly below 1 for X^S−^ and X^D−^. In other words and surprisingly, the PL intensity of X^T−^ is more than 4 times larger when we excite with circularly polarized light. We present in the Supplementary Note 2 the same measurements performed on different spots of the sample and showing the same results. We show in the Supplementary Note 3 that this result is independent from the direction of the linearly polarized excitation.

### Electron density and excitation power dependences

In Fig. 2c, d, we show *P*_c_ and *R* measured at the emission peaks of the bright trion doublet as a function of electron density. The very large positive polarization of X^T−^ is nearly constant while for X^S−^ it varies from positive at small doping to negative for densities above 2 × 10^11^ cm^−2^ and reaches the minimum value of −40% for 4 × 10^11^ cm^−2^. Concerning the ratio of PL intensities between circular and linear excitations (Fig. 2d), it remains above *R* = 2 in the whole investigated electron density range for X^T−^ and slightly below 1 for X^S−^. Finally, we present in Fig. 2e, f the excitation power dependence at an electron doping density of 4 × 10^11^ cm^−2^. We clearly see that when we reduce the excitation power, *P*_c_ converges to a value around 50% for both X^T−^ and X^S−^ and that *R* decreases and gets closer to 1 for X^T−^, while it stays constant and close to 1 for X^S−^.

## Discussion

In the following, we will tentatively explain these results focusing on three clear observations:

(i) the circular polarization of the triplet trion can reach very high positive values.
(ii) the circular polarization of the triplet and singlet trions are of opposite sign at sufficiently large doping level.
(iii) the intensity of the triplet trion is more than 4 times larger with circular excitation than with linear excitation.

**Fig. 3:**
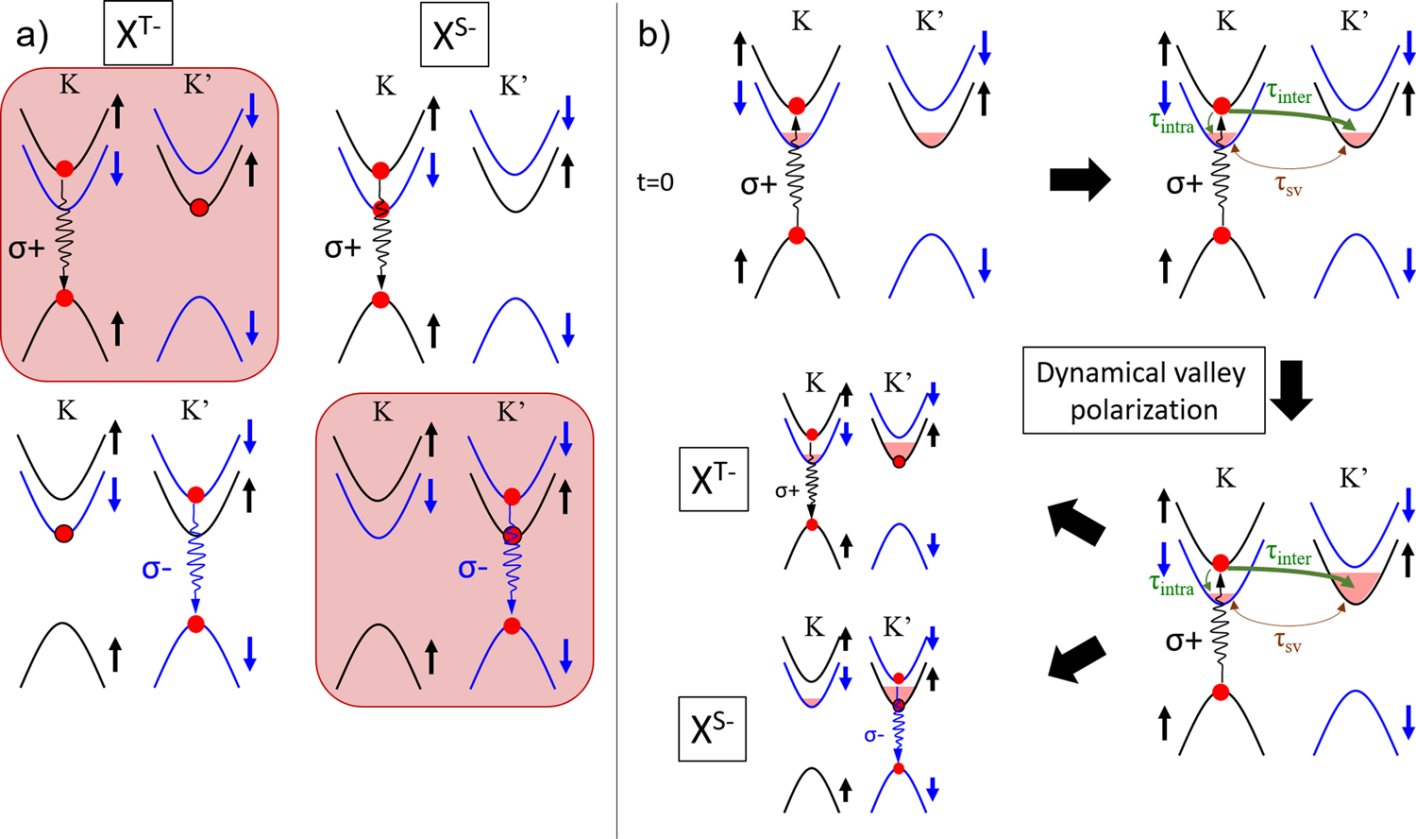
Spin/valley pumping mechanism. **a** Sketches of the three-particle pictures of bright triplet X^T−^ and singlet X^S−^ negative trions with σ+ and σ− emission. The two majority trions following a σ+ cw-excitation are highlighted in red. **b** Sketch of the dynamic polarization of resident electrons with σ+ excitation. *τ*_inter_ (*τ*_intra_) represents the intervalley spin-conserving (intravalley spin-flip) relaxation times of topmost electrons while *τ*_sv_ corresponds to the spin/valley relaxation time of resident electrons.

We show in Fig. 3 the three-particle configurations of σ+ and σ− triplet and singlet trions. A triplet trion consists of a photo-generated electron-hole pair (exciton made of an electron in the topmost conduction band and a missing electron in the same valley) bound to a resident electron in the bottom conduction band lying in the other valley. On the other hand, a singlet trion is composed of a photo-generated electron-hole pair bound to a resident electron in the same valley. Experimentally, we observe that when exciting with a σ+ polarized laser the two strongest PL peaks are the σ+ triplet trion and the σ− singlet trion (Fig. 2a). These two configurations are highlighted in red in Fig. 3a. In both cases the resident electron in the three-particle complex lies in the K′ valley. Thus if we assume that the formation mechanisms of triplet and singlet trions are the same, the opposite sign of polarization of triplet and singlet trions can only be explained by a larger population of resident electrons in the K′ valley as compared to the K valley; i.e. by spin-valley pumping of resident electrons with spin up in K′ valley using σ+ polarized light.

We propose in Fig. 3b a mechanism that dynamically polarizes the resident electrons, in a manner analogous to dynamic spin polarization in conventional semiconductors^38–40^. Without light excitation and magnetic field, the densities of resident electrons are similar in the K and K′ valleys. By continuously exciting with σ+ polarized light at *t* > 0, electrons are photo-generated in the conduction-band top valley of K. These electrons are either free or bound to photo-generated holes and can relax to the bottom conduction bands both in K and K′ valleys through different mechanisms. Intravalley relaxation (time constant τ_intra_ in Fig. 3b) requires an electron spin-flip whereas intervalley relaxation (time constant τ_inter_ in Fig. 3b) conserves the electron’s spin. When energy relaxation is governed by electron–phonon interactions, spin-conserving relaxation is associated to the gradient of the spin-independent component of the crystal potential, while spin-flip interactions are associated to the spin–orbit interaction component. As long as the electronic states are not strongly spin-mixed, the spin-conserving processes are typically stronger, resulting in faster momentum relaxation compared with spin relaxation (i.e. τ_inter_ < τ_intra_). Recently, He et al. analyzed the dark trions’ polarization in ML-WSe_2_ and showed that spin-conserving intervalley relaxation, mediated by zone-edge phonons, is indeed stronger than spin-flip intravalley relaxation that is mediated by zone-center phonons^34^. Consequently, the electron population in the bottommost K′ conduction band becomes larger than the electron population in the bottommost K conduction band upon excitation by a circularly polarized light σ+ (i.e., valley polarization). Under cw-excitation, the mechanism of Fig. 3b is repeated multiple times resulting in a dynamical buildup of valley polarization. This dynamical valley polarization is sustainable if the generation rate of photo-excited electron–hole pairs is faster than the rate at which electrons reestablish thermal equilibrium between the bottommost conduction-band valleys of K and K′ through intervalley spin-relaxation (the spin-valley relaxation time τ_sv_ in Fig. 3b). The latter is a relatively slow process at low temperatures, measured to be as long as 100’s ns to several µs in ML-WSe_2_^22–25^, because it is mediated by spin-flip intervalley transitions, which are forbidden to leading order by time-reversal symmetry^41,42^. Thus, even if the buildup of dynamical valley polarization is slow because the intervalley spin-conserving relaxation (τ_inter_) is not much faster than the intravalley spin-flip one (τ_intra_), the attainable valley polarization can still be very large (we will give an estimate of ~−80% in the following). We mention that other mechanisms of polarization transfer from photo-generated carriers to resident electrons have been proposed by Ersfeld et al^43^. and Fu et al^44^. considering differences in the recombination rates of indirect excitons and spin-forbidden dark excitons or differences in the relaxation rates of singlet and triplet to the dark trions. In each scenario, the two ingredients are the same: creation of an asymmetry in the population of resident electrons and long spin-flip intervalley relaxation times.

Once we consider that resident electrons mainly populate the K′ valley, we can explain the very large polarization of the triplet trion and the negative polarization of the singlet trion. We first assume that the bright trions are formed through the binding of photo-generated bright excitons with a resident electron (i.e. a bimolecular formation^45^). We will discuss other possible mechanisms in a next section and in the Supplementary Information. We also assume that the electron density is much larger than the photo-generated exciton density (see Supplementary Note 4 for our estimation of the exciton density) and we assume that the spin relaxation of trions is much slower than their recombination lifetimes (i.e. the observed polarization in cw experiments correspond to the polarization at the trion formation, see Supplementary Note 5 for the justification of this assumption). In this case we can calculate the polarization of triplet and singlet trions as a function of the polarization of resident electrons ${P}_{{{{{{\mathrm{e}}}}}}}=\frac{{n}_{{{{{{\mathrm{e}}}}}}}^{{{{{{\mathrm{K}}}}}}}-{n}_{{{{{{\mathrm{e}}}}}}}^{{{{{{{\mathrm{K}}}}}}}^{{\prime} }}}{{n}_{{{{{{\mathrm{e}}}}}}}^{{{{{{\mathrm{K}}}}}}}+{n}_{{{{{{\mathrm{e}}}}}}}^{{{{{{{\mathrm{K}}}}}}}^{{\prime} }}}$ (where ${n}_{{{{{{\mathrm{e}}}}}}}^{{{{{{\mathrm{K}}}}}}}$ and ${n}_{{{{{{\mathrm{e}}}}}}}^{{{{{{{\mathrm{K}}}}}}}^{{\prime} }}$ are the populations of resident electrons in the K and K′ valleys) and the polarization of photo-generated excitons ${P}_{0}=\frac{{N}_{0}^{{{{{{\mathrm{K}}}}}}}-{N}_{0}^{{{{{{{\mathrm{K}}}}}}}^{{\prime} }}}{{N}_{0}^{{{{{{\mathrm{K}}}}}}}+{N}_{0}^{{{{{{{\mathrm{K}}}}}}}^{{\prime} }}}$ (where ${N}_{0}^{{{{{{\mathrm{K}}}}}}}$ and ${N}_{0}^{{{{{{{\mathrm{K}}}}}}}^{{\prime} }}$ are the populations of photo-generated excitons in the K and K′ valleys) (see Supplementary Note 6 for more details):

1 $${\underline{{{{{\mathrm{Triplet}}}}}}}\quad\qquad{P}_{{{{{{\mathrm{c}}}}}}}({X}^{T-})=\frac{{P}_{0}-{P}_{{{{{{\mathrm{e}}}}}}}}{1-{P}_{0}{P}_{{{{{{\mathrm{e}}}}}}}}$$

2 $${\underline{{{{{\mathrm{Singlet}}}}}}}\quad\qquad{P}_{{{{{{\mathrm{c}}}}}}}({X}^{S-})=\frac{{P}_{0}+{P}_{{{{{{\mathrm{e}}}}}}}}{1+{P}_{0}{P}_{{{{{{\mathrm{e}}}}}}}}$$

The results of Fig. 2a (*P*_c_(X^T−^) = 91% and (*P*_c_(X^S−^) = −40%) match well with *P*_0_ = 51% and *P*_e_ = −76%; i.e. the resident electrons are strongly polarized in the K′ valley.

This simple scenario of dynamic polarization of electrons is consistent with the power dependence of Fig. 2e. Indeed, when the excitation power decreases, the polarization of both X^T−^ and X^S−^ converge to the same value of around +50%. In this case, the photo-generation rate of electrons is not sufficient to create a significant polarization of resident electrons. Thus the polarizations of X^T−^ and X^S−^ mainly reflect the polarization of the exciton reservoir just before the formation of trions (i.e. the polarization of the hot excitons *P*_0_). Furthermore, the doping density dependence of trions circular polarization of Fig. 2c can be qualitatively explained. For doping densities above 4 × 10^11^ cm^−2^, the polarization in absolute value of both X^T−^ and X^S−^ drops because the density of photo-generated electrons is not large enough to fully polarize the resident electrons. In Supplementary Note 7, we show that increasing the excitation power results in larger polarizations for larger doping densities.

We now discuss the third main result of this work which is another consequence of the efficient spin-valley pumping of resident electrons: the PL intensity of the triplet trion is stronger with circular excitation than with linear excitation (Fig. 2b). Note that this characteristic has been observed in GaAs-based alloys (GaAsN, GaAlAs) where it was attributed to spin dependent recombination via paramagnetic centers^46–48^. Here we attribute it to the efficient spin-valley pumping of resident electrons. Considering the same simple model based on the bimolecular formation of trions that we used to calculate the degrees of circular polarization, we can show that the ratio of PL intensities between circular and linear excitation are (details of the calculations are presented in the Supplementary Note 6):

3 $${\underline{{{{{\mathrm{Triple}}}}}t}}\quad\qquad R({X}^{T-})=1-{P}_{0}{P}_{{{{{{\mathrm{e}}}}}}}$$

4 $${\underline{{{{{\mathrm{Singlet}}}}}}}\quad\qquad R({X}^{S-})=1+{P}_{0}{P}_{{{{{{\mathrm{e}}}}}}}$$

Using the values *P*_0_ = 51% and *P*_e_ = −76% as determined previously we get qualitative agreement with our experimental results: the PL intensity of the triplet trion is larger with circular excitation (i.e. *R*(X^T−^) = 1.39 > 1) and the PL intensity of the singlet trion is larger with linear excitation *R*(X^S−^) = 0.61 < 1.

Nevertheless, our simple model does not describe three quantitative aspects:

(i) the ratio of the total intensities (triplet + singlet: *R*(X^T−^) + X^S−^ using this model is equal to 1 while it is clearly >1 in Fig. 2b.

(ii) *R*(X^*T−*^) cannot be >2 in Eq. (3) while it is experimentally >4.

(iii) The circular polarization of the singlet trion turns positive at low doping in Fig. 2c.

The first limitation suggests that considering the subspace triplet+singlet is insufficient to fully explain our results (i.e. we have a deficit of luminescence for linear excitation). As shown in Fig. 2b, we clearly see that the ratio *R*(*X*^D−^) for the dark trion transition is also below 1; i.e. more intensity with linear excitation than with circular excitation. The dark trion formation path should thus be included.

The two other limitations (ii) and (iii) suggest alternative mechanisms for the formation of the trion species (bright and dark). Theoretical and experimental studies on the trion formation processes in TMD MLs are very scarce. Singh et al. measured the bright trion formation time in MoSe_2_ ML using resonant excitation at the bright exciton transition energy^49^ and linked it to the exciton-electron interaction. Here we use non-resonant excitation above the free carrier band gap of WSe_2_. We can thus propose different formation mechanisms. The bright trions can be formed through the binding of bright excitons with resident electrons (i.e. the bimolecular process already considered above) but also through the binding of two electrons and a hole (i.e. a trimolecular process). In addition, singlet and triplet trions can be formed through the binding of a topmost conduction band electron with respectively a spin-forbidden dark exciton and a momentum-indirect exciton. Similar mechanisms should also be considered for the formation of dark trions in addition to the possible relaxation from bright trions. The dominant formation processes certainly depends on the doping density. For instance the trimolecular process has been demonstrated as dominant in GaAs quantum wells at sufficiently high doping while the bimolecular one is dominant at lower doping densities^45^. The determination of the trion formation processes is beyond the scope of this paper and will require additional theoretical work. In the Supplementary Note 10, we tentatively present a scenario based on trimolecular formation of bright and dark trions that matches quantitatively with the measured values of *P*_c_(X^T−^), *P*_c_(X^S−^), *R*(X^T−^), and *R*(X^S−^) at a doping density of 4 × 10^11^ cm^−2^ and propose some scenarios to explain the positive circular polarization of the singlet trion at low doping.

**Fig. 4:**
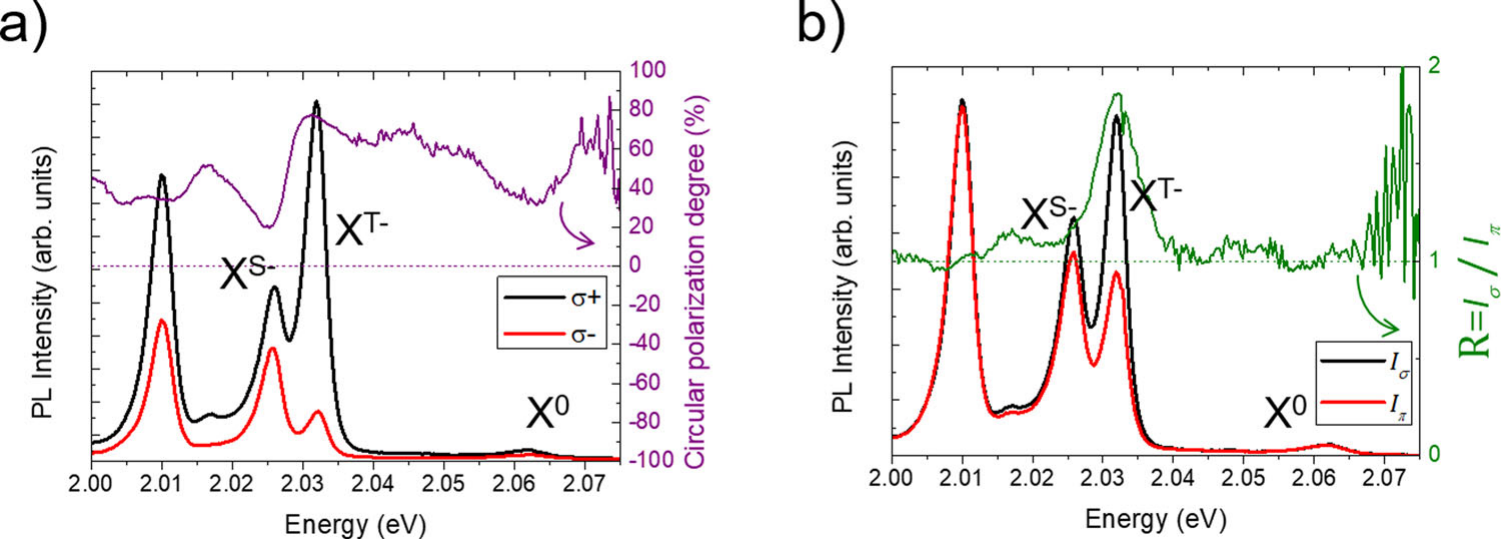
Experimental observations in n-WS_2_. **a** Photoluminescence spectra of WS_2_ for σ+ and σ- detection with σ+ excitation. **b** Photoluminescence spectra of WS_2_ with circular excitation and linear excitation. Excitation power is 18 µW.

Finally, we show that the manifestations of efficient spin valley-pumping of resident electrons in WSe_2_ are also observed in WS_2_. In Fig. 4, we show the PL spectra, the circular polarization degree and the ratio $R=\frac{{I}_{\sigma }}{{I}_{\pi }}$ for a hBN-encapsulated WS_2_ ML. In this case, the ML is not gated but it is intrinsically slightly n-doped as proved by the presence of both triplet and singlet negative trions (X^T−^ and X^S−^) in the luminescence spectra^50–52^. The results are very similar to slightly n-doped WSe_2_: X^T−^ is strongly positively polarized (76% at the peak) and more intense with circular excitation than with linear excitation *R*(X^T−^) = 1.85. We do not observe the negative polarization for the singlet as in Fig. 2a but *P*_c_(X^S−^) is slightly positive as observed for WSe_2_ at smaller doping (Fig. 2c). Similar power dependence is also observed and presented in the Supplementary Note 11.

In summary, we demonstrated very efficient spin-valley pumping of resident electrons in both WSe_2_ and WS_2_ monolayers using circularly polarized light. This process manifests as a large positive circular polarization of the triplet trion, a negative polarization of the singlet trion and a large increase of the triplet trion PL intensity with circular excitation as compared to linear excitation. Interestingly, these results demonstrate that circularly polarized excitation photo-generates electron-hole pairs in one valley and dynamically polarize resident electrons in the opposite valley. This work is thus an important step towards the development of valleytronic devices based on TMD MLs.

## Methods

### Sample fabrication

We have fabricated a van der Waals heterostructure (sketched in Fig. 1a) made of an exfoliated ML-WSe_2_ embedded in high quality hBN crystals^53^ using a dry stamping technique^54^ in the inert atmosphere of a glove box. The layers are deterministically transferred on top of a SiO_2_/Si substrate with Ti/Au electrodes patterned by photolithography. Flux-grown WSe_2_ bulk crystals are purchased from 2D semiconductors. We use few layers of graphene exfoliated from a HOPG bulk crystal for the back gate and to contact the ML-WSe_2_. The WS_2_ sample is fabricated using the same technique but without electrodes.

### Experimental setup

Polarization dependent photoluminescence experiments are performed in close cycle cryostats (*T* = 4 K for WSe_2_ and *T* = 20 K for WS_2_) with diffracted limited laser spot and continuous wave (cw) excitation. For WSe_2_ we use the 632.8 nm line of a HeNe laser while a cw dye laser at a wavelength of 570 nm is used for WS_2_. Polarization measurements are performed using a combination of Glan-Laser polarizers, quarter wave plate, and half wave plate. The signal is dispersed by a monochromator and detected by a CCD camera.

The time-resolved photoluminescence (TRPL) measurements presented in the Supplementary Information are performed in similar conditions: we used a ps-pulsed laser (TiSa) at a wavelength of 695 nm for WSe_2_, and an OPO at 570 nm for WS_2_. The signal is detected by a Hamamatsu streak camera with a time resolution of ~2–3 ps.

## Supplementary information

Supplementary Information

## Data Availability

The data that support the findings of this study are available from the corresponding author upon request.

## References

[1] Mueller T, Malic E (2018). Exciton physics and device application of two-dimensional transition metal dichalcogenide semiconductors. npj 2D Mater. Appl..

[2] Mak KF, Lee C, Hone J, Shan J, Heinz TF (2010). Atomically thin MoS2: a new direct-gap semiconductor. Phys. Rev. Lett..

[3] Splendiani A (2010). Emerging photoluminescence in monolayer MoS2. Nano Lett..

[4] Kormányos A (2015). K·p theory for two-dimensional transition metal dichalcogenide semiconductors. 2D Materials.

[5] Wang G (2018). Colloquium: excitons in atomically thin transition metal dichalcogenides. Rev. Mod. Phys..

[6] Xiao D, Liu G-B, Feng W, Xu X, Yao W (2012). Coupled spin and valley physics in monolayers of MoS2 and other group-VI dichalcogenides. Phys. Rev. Lett..

[7] Xu X, Yao W, Xiao D, Heinz TF (2014). Spin and pseudospins in layered transition metal dichalcogenides. Nat. Phys..

[8] Schaibley JR (2016). Valleytronics in 2D materials. Nat. Rev. Mater..

[9] Mak KF, Xiao D, Shan J (2018). Light–valley interactions in 2D semiconductors. Nat. Photon..

[10] Zeng H, Dai J, Yao W, Xiao D, Cui X (2012). Valley polarization in MoS 2 monolayers by optical pumping. Nat. Nanotechnol..

[11] Cao T (2012). Valley-selective circular dichroism of monolayer molybdenum disulphide. Nat. Commun..

[12] Mak KF, He K, Shan J, Heinz TF (2012). Control of valley polarization in monolayer MoS2 by optical helicity. Nat. Nanotechnol..

[13] Sallen G (2012). Robust optical emission polarization in MoS 2 monolayers through selective valley excitation. Phys. Rev. B.

[14] Robert C (2016). Exciton radiative lifetime in transition metal dichalcogenide monolayers. Phys. Rev. B.

[15] Moody G, Schaibley J, Xu X (2016). Exciton dynamics in monolayer transition metal dichalcogenides. J. Opt. Soc. Am. B.

[16] Palummo M, Bernardi M, Grossman JC (2015). Exciton radiative lifetimes in two-dimensional transition metal dichalcogenides. Nano Lett..

[17] Zhu CR (2014). Exciton valley dynamics probed by Kerr rotation in WSe2 monolayers. Phys. Rev. B.

[18] Yu T, Wu MW (2014). Valley depolarization due to intervalley and intravalley electron-hole exchange interactions in monolayer MoS2. Phys. Rev. B.

[19] Jiang C (2018). Microsecond dark-exciton valley polarization memory in two-dimensional heterostructures. Nat. Commun..

[20] Rivera P (2016). Valley-polarized exciton dynamics in a 2D semiconductor heterostructure. Science.

[21] Qu F (2019). Controlling valley splitting and polarization of dark- and bi-excitons in monolayer WS2 by a tilted magnetic field. 2D Materials.

[22] Dey P (2017). Gate-controlled spin-valley locking of resident carriers in WSe2 monolayers. Phys. Rev. Lett..

[23] Goryca M, Wilson NP, Dey P, Xu X, Crooker SA (2019). Detection of thermodynamic “valley noise” in monolayer semiconductors: access to intrinsic valley relaxation time scales. Sci. Adv..

[24] Yan T, Yang S, Li D, Cui X (2017). Long valley relaxation time of free carriers in monolayer WSe2. Phys. Rev. B.

[25] Li J (2021). Valley relaxation of resident electrons and holes in a monolayer semiconductor: dependence on carrier density and the role of substrate-induced disorder. Phys. Rev. Mater..

[26] Back P (2017). Giant paramagnetism-induced valley polarization of electrons in charge-tunable monolayer MoSe2. Phys. Rev. Lett..

[27] Glazov MM (2020). Optical properties of charged excitons in two-dimensional semiconductors. J. Chem. Phys..

[28] Sidler M (2017). Fermi polaron-polaritons in charge-tunable atomically thin semiconductors. Nat. Phys..

[29] Jones AM (2016). Excitonic luminescence upconversion in a two-dimensional semiconductor. Nat. Phys..

[30] Courtade E (2017). Charged excitons in monolayer WSe2: experiment and theory. Phys. Rev. B.

[31] Wang G (2017). In-plane propagation of light in transition metal dichalcogenide monolayers: optical selection rules. Phys. Rev. Lett..

[32] Zhang X-X (2017). Magnetic brightening and control of dark excitons in monolayer WSe2. Nat. Nanotechnol..

[33] Zhou Y (2017). Probing dark excitons in atomically thin semiconductors via near-field coupling to surface plasmon polaritons. Nat. Nanotechnol..

[34] He M (2020). Valley phonons and exciton complexes in a monolayer semiconductor. Nat. Commun..

[35] Li Z, Wang T, Miao S, Lian Z, Shi S-F (2020). Fine structures of valley-polarized excitonic states in monolayer transitional metal dichalcogenides. Nanophotonics.

[36] Liu E (2019). Gate tunable dark trions in monolayer WSe2. Phys. Rev. Lett..

[37] Li Z (2019). Direct observation of gate-tunable dark trions in monolayer WSe2. Nano Lett..

[38] Meier, F. & Zakharchenya, B. P. *Optical Orientation* (Elsevier, 1984).

[39] Kikkawa JM, Awschalom DD (1998). Resonant spin amplification in n-type GaAs. Phys. Rev. Lett..

[40] Childress L (2006). Coherent dynamics of coupled electron and nuclear spin qubits in diamond. Science.

[41] Song Y, Dery H (2013). Transport theory of monolayer transition-metal dichalcogenides through symmetry. Phys. Rev. Lett..

[42] Dery H, Song Y (2015). Polarization analysis of excitons in monolayer and bilayer transition-metal dichalcogenides. Phys. Rev. B.

[43] Ersfeld M (2020). Unveiling valley lifetimes of free charge carriers in monolayer WSe2. Nano Lett..

[44] Fu J, Cruz JMR, Qu F (2019). Valley dynamics of different trion species in monolayer WSe2. Appl. Phys. Lett..

[45] Portella-Oberli MT (2009). Dynamics of trion formation in InxGa1-xAs quantum wells. Phys. Rev. Lett..

[46] Weisbuch C, Lampel G (1974). Spin-dependent recombination and optical spin orientation in semiconductors. Solid State Commun..

[47] Paget D (1984). Optical-pumping study of spin-dependent recombination in GaAs. Phys. Rev. B.

[48] Lagarde D (2007). Electron spin dynamics in GaAsN and InGaAsN structures. Phys. Status Solidi A.

[49] Singh A (2016). Trion formation dynamics in monolayer transition metal dichalcogenides. Phys. Rev. B.

[50] Plechinger G (2016). Trion fine structure and coupled spin–valley dynamics in monolayer tungsten disulfide. Nat. Commun..

[51] Vaclavkova D (2018). Singlet and triplet trions in WS2 monolayer encapsulated in hexagonal boron nitride. Nanotechnology.

[52] Paur M (2019). Electroluminescence from multi-particle exciton complexes in transition metal dichalcogenide semiconductors. Nat. Commun..

[53] Cadiz F (2017). Excitonic linewidth approaching the homogeneous limit in MoS2-based van der Waals heterostructures. Phys. Rev. X.

[54] Castellanos-Gomez A (2014). Deterministic transfer of two-dimensional materials by all-dry viscoelastic stamping. 2D Materials.

