# Supplementary material for: Spin/valley pumping of resident electrons in WSe2 and WS2 monolayers
Source: Nat Commun. 2021 Sep 15;12:5455. doi: 10.1038/s41467-021-25747-5 (PMC8443707; doi:10.1038/s41467-021-25747-5)
Supplement: Supplementary file 1 — Supplementary Information [file 41467_2021_25747_MOESM1_ESM.pdf]

Supplementary Information for "Spin/Valley pumping of resident electrons in WSe_2 and WS_2 monolayers"

Cedric Robert[1], Sangjun Park[2], Fabian Cadiz[2], Laurent Lombez[1], Lei Ren[1], Hans Tornatzky[1], Alistair Rowe[2], Daniel Paget[2], Fausto Sirotti[2], Min Yang[3], Dinh Van Tuan[3], Takashi Taniguchi[4], Bernhard Urbaszek[1], Kenji Watanabe[5], Thierry Amand[1], Hanan Dery[3,6] and Xavier Marie[1]

[1]Université de Toulouse, INSA-CNRS-UPS, LPCNO, 135 Av. Rangueil, 31077 Toulouse, France
[2]Physique de la matière condensée, Ecole Polytechnique, CNRS, IP Paris, 91128 Palaiseau, France
[3]Department of Electrical and Computer Engineering, University of Rochester, Rochester, New York 14627, USA
[4]International Center for Materials Nanoerchitectonics, National Institute for Materials Science, 1-1 Namiki, Tsukuba 305-00044, Japan
[5]Research Center for Functional Materials, National Institute for Materials Science, 1-1 Namiki, Tsukuba 305-00044, Japan
[6]Department of Physics, University of Rochester, Rochester, New York 14627, USA

Supplementary Note 1. Estimation of the carrier density

The carrier density in the charge tunable device is estimated by two methods as in our previous work [1]. The first one uses the simple plate capacitance model. Knowing the applied voltage (V), the hBN thickness t (210 nm in our device) and using a hBN dielectric constant of ε_{hBN}~3 [2,3], the change of electron density Δn is related to a change of bias voltage ΔV by $\Delta n = \frac{\varepsilon_0 \varepsilon_{hBN}}{e*t} \Delta V$. Alternatively, we can use the oscillations in the reflectivity spectrum of the bright exciton as a function of gate voltage in the p-doped regime observed at +9 T (see Supplementary Figure S1(b)). As demonstrated in Ref [4], these oscillations are due to the interaction of the exciton with the quantized Landau levels of the hole Fermi sea (see the sketch of Supplementary Figure S1(a)). The period of the oscillations ΔV_{LL} is related to the filling of one Landau level $P_{LL} = \frac{eB}{2\pi\hbar}$ =2.18 10^{11} cm^{-2}. We can thus calculate the hole density as a function of the gate voltage by: $\Delta p = \Delta V \frac{P_{LL}}{\Delta V_{LL}}$. This yields the same estimation of the carrier density as the one deduced from the capacitance model. The advantage of this method is that it does not require knowledge of material parameters.

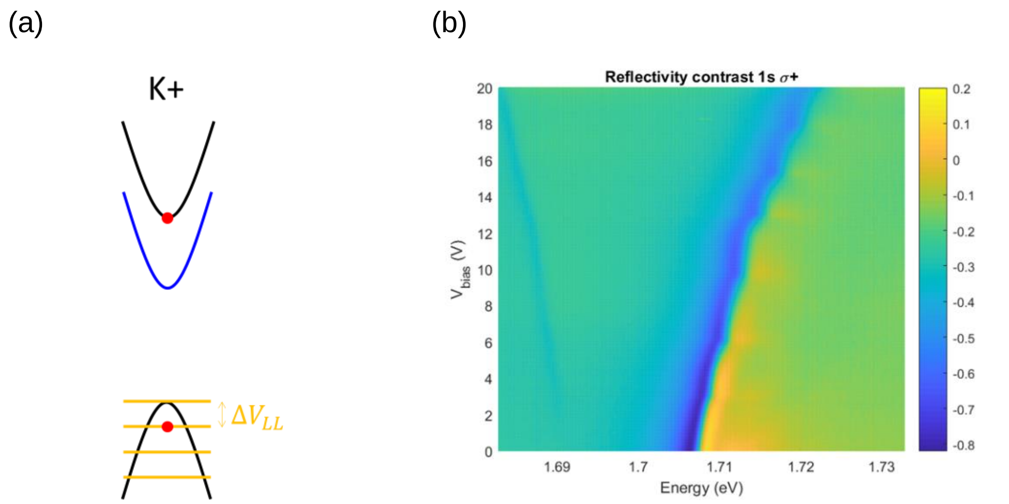

Supplementary Figure S1: Sketch of the band structure of the K+ valley at positive magnetic field showing the Landau levels. (b) Reflectivity contrast of the bright exciton with σ+ detection as a function of the gate voltage at +9 T

Supplementary Note 2. Additional measurements on other spots of the sample

We reproduced the measurements of Figure 2a and 2b of the main text on different spots of the sample. On each spot we find a very large circular polarization of the triplet trion, a negative circular polarization of the singlet trion and a PL intensity for the triplet trion more than 4 times larger with circular excitation than with linear excitation.

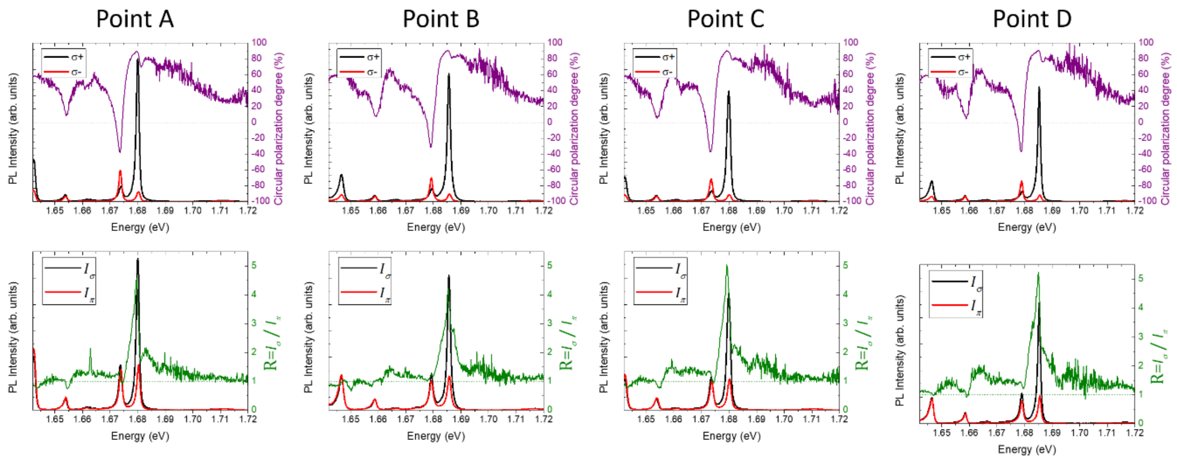

Supplementary Figure S2: Photoluminescence and circular polarization spectra for σ+ and σ- detections with σ+ excitation and total photoluminescence spectra with circular excitation and linear excitation on 4 different spots of the sample. The excitation power is 5 µW and the doping density is 4x10^{11} cm^{-2}.

Supplementary Note 3. PL Intensity as a function of the direction of the linearly polarized excitation

We checked that the PL intensity of both triplet and singlet trions does not depend on the direction of the linearly polarized excitation with respect to the sample excluding any possible role of strain or defects on our interpretation. Results are shown in Supplementary Figure S3.

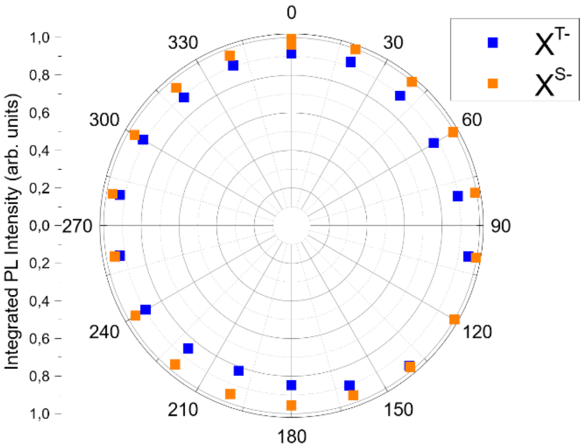

Supplementary Figure S3: Integrated intensities of the triplet and singlet trions as a function of the angle of the linear excitation. Excitation power is 5 µW and the doping density is 4x10^{11} cm^{-2}.

Supplementary Note 4. Estimation of the density of photo-generated electron-hole pairs

We can estimate the density of photo-generated bright electron-hole pairs (or excitons) from the excitation power density, the absorption coefficient and the lifetime of photo-generated electron-hole pairs. We have:

$$N_X = \frac{P_S \times \tau \times \alpha}{E_{photon}}$$

where P_S is the excitation power density (500 W.cm^{-2} for a power of 5 µW and a spot size of 1 µm^2), E_{photon}=1.96 eV for an excitation wavelength of 633 nm. A significant uncertainty comes from the absorption coefficient. For this non resonant wavelength, α was measured for free standing WSe_2 ML in the range 2-3% [2]. Assuming a lifetime τ of ~1 ps, this gives N_X~4x10^7 cm^{-2}, thus much smaller than the doping density.

Supplementary Note 5. Lifetime and spin relaxation time of trions

The degree of circular polarization of a state in a cw experiment is usually given by:

$$P = \frac{P_G}{1 + \frac{\tau}{\tau_S}}$$

where P_G is the degree of circular polarization at the generation, τ is the lifetime of the state and τ_S is the spin (or valley) relaxation time (assuming here a single relaxation mechanism). In the simple model presented in the main text, we considered that the measured degrees of circular polarization for the triplet and the singlet directly reflect P_G. In other words we disregard the role of τ and τ_S. We give some experimental justifications below. We performed time-resolved photoluminescence in a second WSe_2 charge tunable device and in the same WS_2 monolayer that is presented in the main text. In WSe_2 we measured the lifetime of both triplet and singlet as function of the doping density and in WS_2 we measured the decay of the circular polarization. Results are presented in Supplementary Figure S4. At very low doping regime (~1x10^{11} cm^{-2}), the triplet and the singlet luminescence decay with a lifetime τ of the order of 10-20 ps. When the doping increases to a few 10^{11} cm^{-2}, the decay is strongly reduced to ~1 ps. We can have an idea of the spin relaxation time of trions τ_S by looking at the decay of the circular polarization of the triplet in WS_2 (Supplementary Figure S4c). Our results suggest that this time is very slow (longer than 200 ps), thus much longer than the lifetime. In conclusion, it is reasonable to assume that $P \approx P_G$.

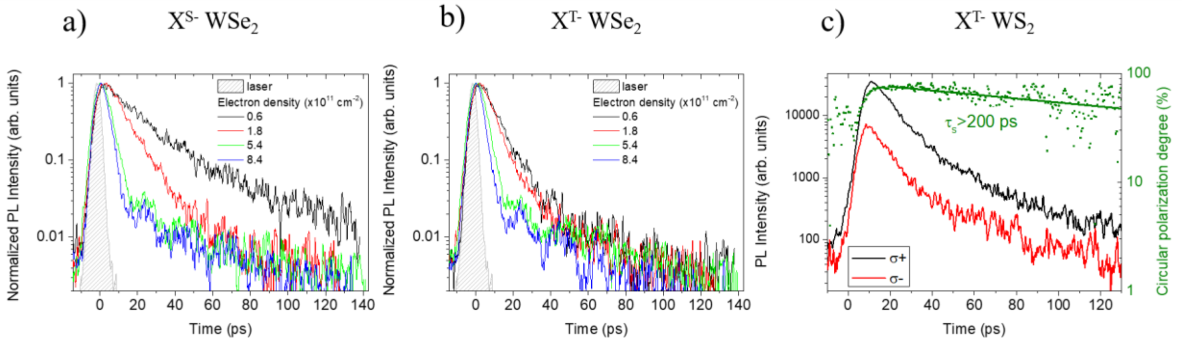

Supplementary Figure S4: Time-resolved photoluminescence measurements of the (a) singlet and (b) triplet in a second WSe_2 charge tunable device showing the shortening of the lifetime when the electron doping increases. (c) Time-resolved photoluminescence measurements of the triplet in the WS_2 monolayer sample of the main text. In this measurement, we excite with σ+ polarized light and detect both σ+ and σ- decays. In green we plot the circular polarization degree as a function of time. It shows that the spin/valley relaxation time of trions is much longer than the lifetime.

Supplementary Note 6. Formation of bright trions through the binding of bright excitons and resident electrons: simple bimolecular model

In a simple bimolecular formation of trions we can write that the population of a given trion configuration $N^{\sigma+,-}_{X^{S,T-}}$ is proportional to the population of the photogenerated bright exciton $N_0^{K,K'}$ times the population of resident electrons $n_e^{K,K'}$. Thus we can write the degree of circular polarization for X^{T-} and X^{S-} as:

$$P_c(X^{T-}) = \frac{N^{\sigma^+}_{X^{T-}} - N^{\sigma^-}_{X^{T-}}}{N^{\sigma^+}_{X^{T-}} + N^{\sigma^-}_{X^{T-}}} \propto \frac{N_0^K n_e^{K'} - N_0^{K'} n_e^K}{N_0^K n_e^{K'} + N_0^{K'} n_e^K} \tag{S1}$$

$$P_c(X^{S-}) = \frac{N^{\sigma^+}_{X^{S-}} - N^{\sigma^-}_{X^{S-}}}{N^{\sigma^+}_{X^{S-}} + N^{\sigma^-}_{X^{S-}}} \propto \frac{N_0^K n_e^K - N_0^{K'} n_e^{K'}}{N_0^K n_e^K + N_0^{K'} n_e^{K'}} \tag{S2}$$

we can also define the degree of polarization of the photo-generated bright exciton (hot photo-generated excitons; or unbound electron-hole pairs; that eventually bind to resident electrons to form trions) as:

$$P_0 = \frac{N_0^K - N_0^{K'}}{N_0^K + N_0^{K'}} \tag{S3}$$

and the degree of polarization of resident electrons as:

$$P_e = \frac{n_e^K - n_e^{K'}}{n_e^K + n_e^{K'}} \tag{S4}$$

Combining the four equations we can show that:

$$P_c(X^{T-}) = \frac{P_0 - P_e}{1 - P_0 P_e} \tag{S5}$$

$$P_c(X^{S-}) = \frac{P_0 + P_e}{1 + P_0 P_e} \tag{S6}$$

Using the same model we can write the total PL intensities of triplet and singlet following circularly polarized excitation as:

$$I_{circ}(X^{T-}) \propto N_{X^{T-}}^{\sigma^+} + N_{X^{T-}}^{\sigma^-} \propto N_0^K n_e^{K'} + N_0^{K'} n_e^K \tag{S7}$$

$$I_{circ}(X^{S-}) \propto N_{X^{S-}}^{\sigma^+} + N_{X^{S-}}^{\sigma^-} \propto N_0^K n_e^K + N_0^{K'} n_e^{K'} \tag{S8}$$

For linear excitation, the total intensity is:

$$I_{lin}(X^{T-}) = I_{lin}(X^{S-}) \propto \frac{1}{2}\left(N_0^K + N_0^{K'}\right)\left(n_e^K + n_e^{K'}\right) \tag{S9}$$

Thus the ratio of intensities between circular and linear excitation simply write:

$$R(X^{T-}) = \frac{I_{circ}(X^{T-})}{I_{lin}(X^{T-})} = 1 - P_0 P_e \tag{S10}$$

$$R(X^{S-}) = \frac{I_{circ}(X^{S-})}{I_{lin}(X^{S-})} = 1 + P_0 P_e \tag{S11}$$

In Supplementary Figure S5, we plot $P_c(X^{T-})$, $P_c(X^{S-})$, $R(X^{T-})$and $R(X^{S-})$ as a function of P_0 and P_e.

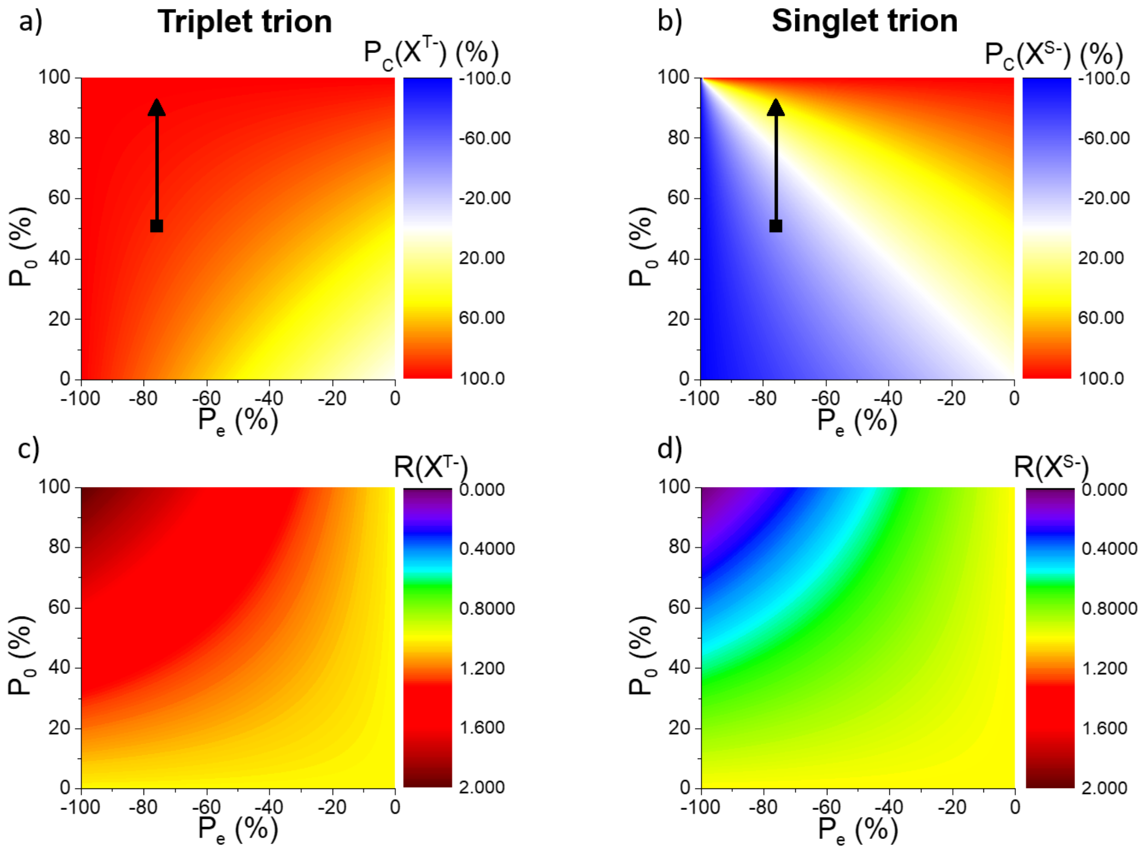

*Supplementary Figure S5: **Bimolecular model**: Calculated degrees of PL circular polarization P_c for (a) triplet and (b) singlet trions as function of the polarization of resident electrons (P_e) and the polarization of photogenerated excitons (P_0). (c) and (d) show the calculated ratio R for both triplet and singlet using the same model. The black data point correspond to the regime of Figure 2a of the main text where $P_c(X^{T-})$=91% and $P_c(X^{S-})=-40\%$. The vertical arrow correspond to the possible increase of P_0 when the doping decreases that may explain the results of Figure 2c where $P_c(X^{S-})$ turns positive and $P_c(X^{T-})$ remains very large.*

Supplementary Note 7. Additional data with increased excitation power

We show here the PL circular polarization degree and the ratio R of both triplet and singlet trions as a function of the doping density for an increased excitation power of 20 µW. As compared to the measurements of Figure 2c and 2d (taken at 5 µW), the minimum of $P_c(X^{S-})$ is shifted above 4×10^{11} cm^{-2}, and the maximum of R(X^{T-}) is found around 5×10^{11} cm^{-2} as compared to 3-4×10^{11} cm^{-2} at 5 µW. The overall shifts toward higher electron density is consistent with our scenario of dynamical polarization of resident electrons.

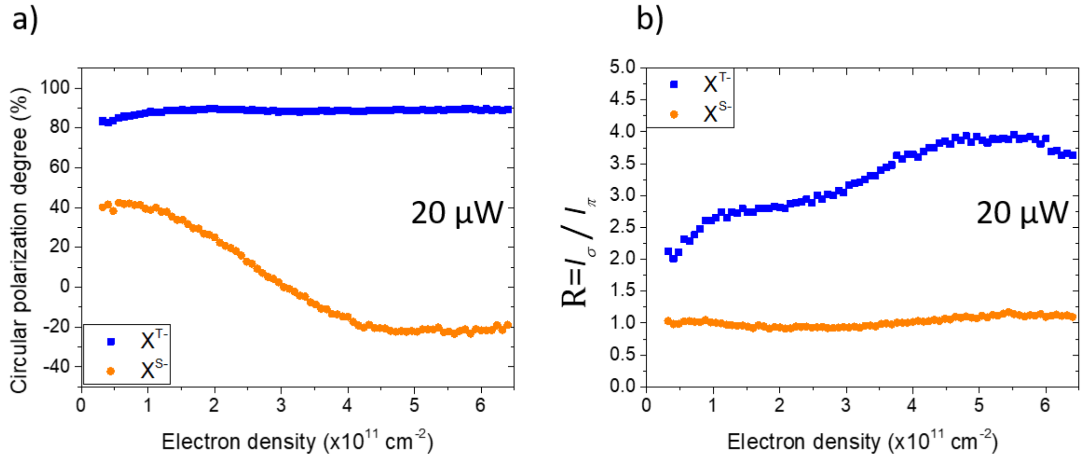

Supplementary Figure S6: (a) Circular polarization degree at the peak of triplet and singlet negative trions as a function of electron density for an excitation power of 20 µW. (b) Ratio of PL intensities between circular and linear excitations at the peak for both triplet and singlet as a function of electron density for an excitation power of 20 µW.

Supplementary Note 8. Possible interpretation of the positive circular polarization of the singlet trion at low doping

In Figure 2c of the main text, we show that when the doping density decreases from 4x10^{11} cm^{-2}, the negative circular polarization of the singlet trion X^{S-} drops and even turns positive below 2x10^{11} cm^{-2}. According to our scenario of dynamical building of polarization of resident electrons through cw circular excitation, there is no reason for the resident electrons to be less polarized at smaller doping densities. On the opposite, we showed that the polarization of resident electrons is favored when the density of photogenerated electrons is sufficient as compared to the doping density (see the power dependence of Figure 2e in the main text). Thus if we assume that the polarization of resident electrons P_e remains at least -76% at low doping (the value we determined at 4x10^{11} cm^{-2} in the main text), our simple bimolecular model dictates that the polarization of X^{S-} can turn positive and the polarization of X^{T-} remains very large if $P_0 > -P_e$ (i.e. P_0 >76%) (see equation (2) of the main text and Supplementary Figure S5). In other words, our results can be explained if the polarization of photo-generated excitons P_0 increases when the doping density decreases.

Note that P_0 does not directly correspond to the polarization of bright excitons X^0 measured in PL ($P_c(X^0)$~15% in Figure2a). Indeed, $P_c(X^0)$ corresponds to cold bright excitons (i.e. radiatively recombining in the light cone) while P_0 corresponds to hot photo-generated excitons (or even unbound electron-hole pairs) that bind to resident electrons to form trions. In a simple approach we can write:

$$P_c(X^0) = \frac{P_0}{1 + \frac{\tau^{X^0}}{\tau_s^{X^0}}}$$

where τ^{X^0} and $\tau_s^{X^0}$ are the bright exciton lifetime and spin relaxation time. When the doping decreases, we can assume that τ^{X^0}remains constant or increases and that $\tau_s^{X^0}$ remains constant or decreases (the long range exchange interaction may be less efficient at higher doping because of the screening by the Fermi sea). In Supplementary Figure S7, we show that $P_c(X^0)$ increases up to 35% when the electron doping decreases. Thus it is consistent with an increase of P_0 when the doping decreases.

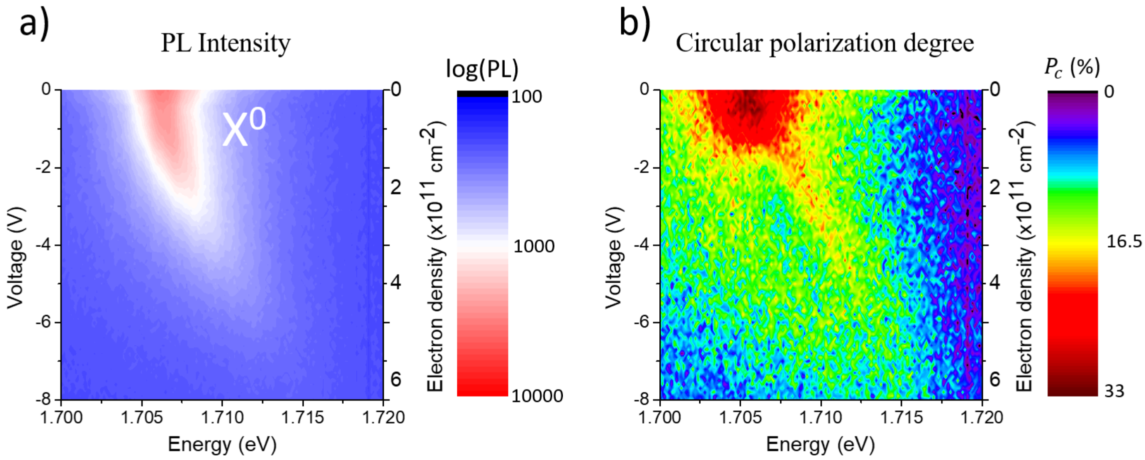

Supplementary Figure S7: (a) Zoom on the PL intensity of the bright exciton X^0 as a function of electron density. (b) Corresponding circular polarization degree showing an increase of $P_c(X^0)$ when the doping decreases.

Supplementary Note 9. Alternative formation mechanisms of trions

In the main text we considered that bright trions are formed through a simple bimolecular mechanism involving a bright exciton and bottommost conduction band electrons. We briefly detail in this section alternative formation mechanisms.

- triplet trions can be formed through the binding of indirect excitons and topmost conduction band electrons.

- singlet trions can be formed through the binding of dark excitons and topmost conduction band electrons.

- triplet trions can convert to singlet trions through a second-order exchange process. The triplet has higher energy than the singlet by Δ_{TS} ~6 meV, and therefore, an electron-hole exchange of the bright exciton component in the trion cannot alone switch between triplet and singlet trion states. However, the conversion from the higher energy trion (triplet) to the lower-energy one (singlet) can proceed if the electron-hole exchange is accompanied by Coulomb scattering of the trion with a cold resident electron. Here, the excess of Δ_{TS} ~6 meV are mostly gained by the resident electron after scattering. The singlet-to-triplet conversion is suppressed because this would require the resident electron to give away 6 meV, which is not possible if the electron is cold (resides in the bottom of the valley).

- triplet trions can become dark trions following emission of a zone-center phonon Γ_5.

- singlet trions can become dark trions following emission of a zone-edge phonon K_2.

- bright trions can turn to dark through trion-electron Coulomb scattering. Here, an incoming free electron kicks the top-valley electron from the incoming bright trion complex, and binds to the left-behind exciton component. The end products are a top-valley electron and a bound dark trion with two electrons in time-reversed bottom valleys. The energy conservation of this process mandates that the top-valley free electron after the scattering gains kinetic energy compared to the kinetic energy of the incoming electron. Importantly, this bright-to-dark trion conversion is suppressed when the free resident electrons are strongly polarized (e.g., resident electrons reside mostly in K' and the bottom-valley electron of the triplet or singlet is also in K'). This mechanism is thus consistent with the positive polarization of the triplet and the negative polarization of the singlet.

- When the electrostatic doping is relatively large, a singlet (triplet) trion may be formed through a trimolecular binding process wherein a photoexcited hole, photoexcited electron (top-valley), and a resident electron (bottom valley) bind to form a bright trion. This process requires a relatively large density of resident electrons in the bottom valleys, so that trions are formed before the top-valley electrons relax to the bottom valleys. This mechanism is detailed in the next section.

Supplementary Note 10. Trimolecular model

We show here that the results of Figure 2a and 2b of the main text can be interpreted using a model taking into account a trimolecular formation of bright (singlet and triplet) and dark negative trions. We detail the model below. We use the notations of Supplementary Figure S8 to describe the populations of electrons, holes and trions in each valley.

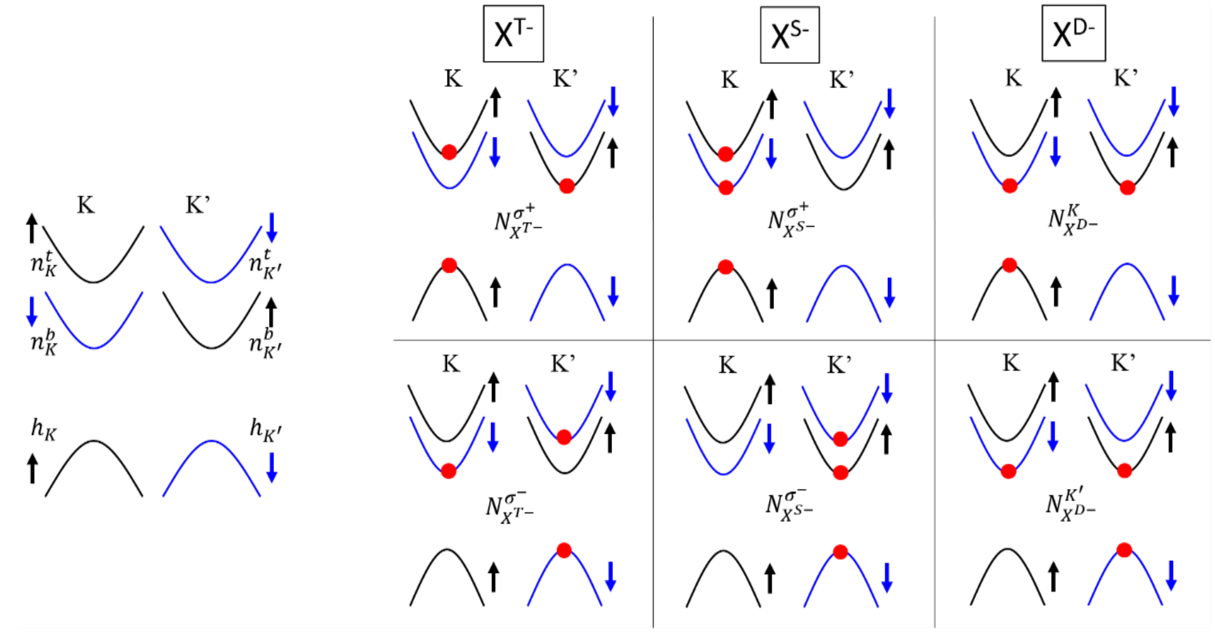

Supplementary Figure S8: Notations of the different electron, hole and trion populations used in the rate equation model. The arrows represent the electron conduction or valence spin states in each valley.

For the sake of simplicity, we consider in our model only the formation and recombination mechanisms of trions (no spin relaxation). This can be easily justified by the very short lifetime of the trions as compared to the spin relaxation times (see Supplementary Figure S4). We also consider that the dynamic polarization of resident electrons takes place at a much slower timescale than the formation and recombination of the trions so that n_K^b and $n_{K'}^b$ are fixed (they are considered as parameters of the model). We have $n_K^b + n_{K'}^b = n_e$ where n_e is the doping density and $P_e = \frac{n_K^b - n_{K'}^b}{n_K^b + n_{K'}^b}$ is the polarization of the resident electrons.

Within these assumptions, the different electron and hole populations satisfy the following system of rate equations:

$$\frac{dn_K^t}{dt} = G_K^0 - C_{triplet} n_K^t n_{K'}^b h_K - C_{singlet} n_K^t n_K^b h_K - \frac{n_K^t}{\tau_0}$$

$$\frac{dn_{K'}^t}{dt} = G_{K'}^0 - C_{triplet} n_{K'}^t n_K^b h_{K'} - C_{singlet} n_{K'}^t n_{K'}^b h_{K'} - \frac{n_{K'}^t}{\tau_0}$$

$$\frac{dh_K}{dt} = G_K^0 - C_{triplet} n_K^t n_{K'}^b h_K - C_{singlet} n_K^t n_K^b h_K - C_{dark} n_K^b n_{K'}^b h_K - \frac{h_K}{\tau_0}$$

$$\frac{dh_{K'}}{dt} = G_{K'}^0 - C_{triplet} n_{K'}^t n_K^b h_{K'} - C_{singlet} n_{K'}^t n_{K'}^b h_{K'} - C_{dark} n_{K'}^b n_K^b h_{K'} - \frac{h_{K'}}{\tau_0}$$

and the different populations of trions read as:

$$\frac{dN_{X^{T-}}^{\sigma^+}}{dt} = C_{triplet} n_K^t n_{K'}^b h_K - \frac{N_{X^{T-}}^{\sigma^+}}{\tau_T}$$

$$\frac{dN_{X^{T-}}^{\sigma^-}}{dt} = C_{triplet} n_{K'}^t n_K^b h_{K'} - \frac{N_{X^{T-}}^{\sigma^-}}{\tau_T}$$

$$\frac{dN_{X^{S-}}^{\sigma^+}}{dt} = C_{singlet} n_K^t n_K^b h_K - \frac{N_{X^{S-}}^{\sigma^+}}{\tau_S}$$

$$\frac{dN_{X^{S-}}^{\sigma^-}}{dt} = C_{singlet} n_{K'}^t n_{K'}^b h_{K'} - \frac{N_{X^{S-}}^{\sigma^-}}{\tau_S}$$

$$\frac{dN_{X^{D-}}^{K}}{dt} = C_{dark} n_K^b n_{K'}^b h_K - \frac{N_{X^{D-}}^{K}}{\tau_D}$$

$$\frac{dN_{X^{D-}}^{K'}}{dt} = C_{dark} n_{K'}^b n_K^b h_{K'} - \frac{N_{X^{D-}}^{K'}}{\tau_D}$$

where $C_{triplet}$, $C_{singlet}$, and C_{dark} are the trimolecular formation rates of X^{T-}, $X^{S-}$and X^{D-}. τ_T, τ_S, τ_D and τ_0 are the lifetimes of X^{T-}, X^{S-}, X^{D-} and X^0. G_K^0, $G_{K'}^0$ are the generation rates of the photogenerated electron-hole pairs in each valley.

- We fix τ_0=1 ps, τ_T=τ_S=10 ps and τ_D=500 ps as measured in time-resolved photoluminescence experiments (see Supplementary Figure S4 and Supplementary Figure S9). Note that except for τ_0, these parameters do not change the values of $P_c(X^{T-})$, $P_c(X^{S-})$, $R(X^{T-})$ and $R(X^{S-})$.
- G_K^0 and $G_{K'}^0$ are defined through the total generation rate of electron-hole pairs $G^0 = \frac{P_S \times \alpha}{E_{photon}}$ and $P_0 = \frac{G_0^K - G_0^{K'}}{G_0^K + G_0^{K'}}$ where α is the absorption coefficient and P_S is the excitation power density.
- We fix $C_{triplet} = C_{singlet} = C_B$ as we expect the formation coefficient of triplet and singlet to be similar given that they have very similar oscillator strength (see reflectivity spectra in Figure 1b of the main text and [3]).

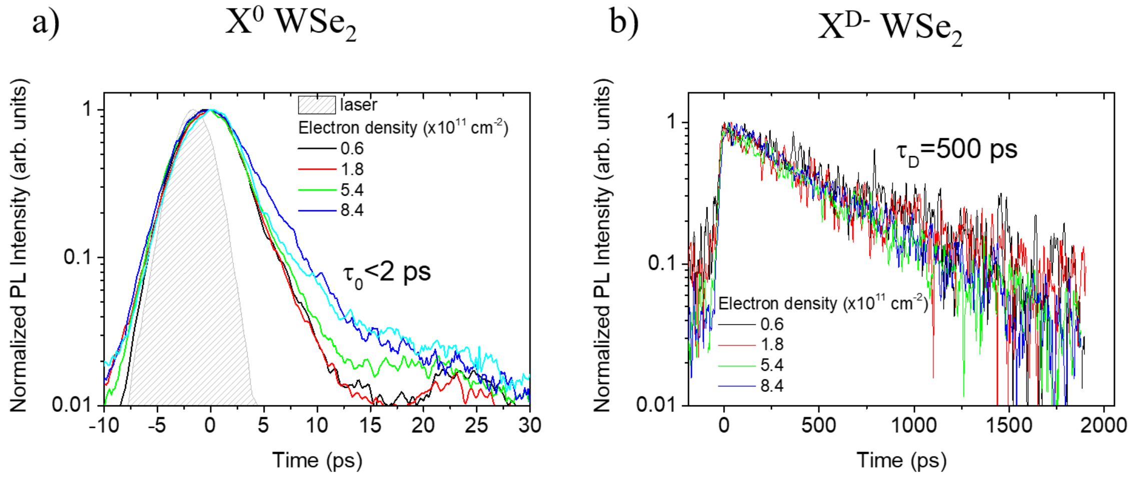

Supplementary Figure S9: Time-resolved photoluminescence measurements of the (a) bright exciton and (b) dark trion in a second WSe_2 charge tunable device. The bright exciton lifetime is close to the temporal resolution. We chose to fix τ_0 in agreement with the values found in the literature [ref].

The different populations for both circular and linear excitations are calculated by solving the equations system in stationary conditions ($\frac{d}{dt}$=0). We fit simultaneously $P_c(X^{T-})$, $P_c(X^{S-})$, $R(X^{T-})$ and $R(X^{S-})$ to the experimental values of Figure 2 of the main text ($P_c(X^{T-})$=91%, $P_c(X^{S-})$=−40%, $R(X^{T-})$=4.4 and $R(X^{S-})$=0.88) by adjusting 5 parameters: P_0, P_e, α, C_B and C_D

We obtain $P_c(X^{T-})$=98%, $P_c(X^{S-})$=−40%, $R(X^{T-})$=4.4 and $R(X^{S-})$=0.9 for P_0=57%, P_e=−88%, α=2.95%, C_B=4.65x10^{-17} cm^{-4}ps^{-1}, C_D=4.8x10^{-20} cm^{-4}ps^{-1}.

P_0 and P_e are in the same range than the values obtained with the simple bimolecular model (P_0=51% and P_e=−76%). We show in Supplementary Figure S10, the influence of these two parameters on the results of the trimolecular model.

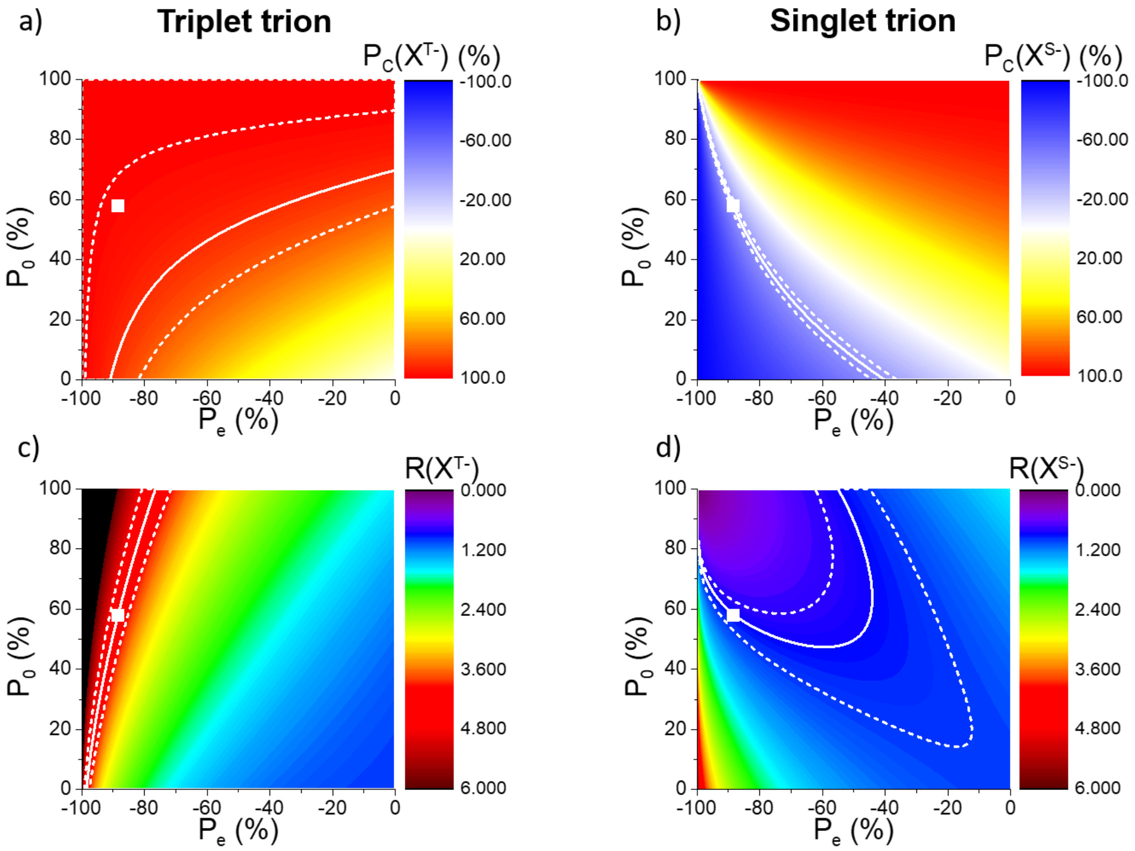

Supplementary Figure S10: ***Trimolecular model****: Influence of P_0 and P_e on the calculated degrees of circular polarization for (a) triplet and (b) singlet trions and on the ratio R (c) and (d). The white data point correspond to the result of the fit (P_0=57%, P_e=-88%). The white solid lines are the contour plot of the experimental data ($P_c(X^{T-})$=91%, $P_c(X^{S-})$=-40%, $R(X^{T-})$=4.4 and $R(X^{S-})$=0.88) and the dashed lines are the contour plots of the experimental data with a tolerance of ±10%. All other parameters are fixed to the values written in the text.*

The absorption coefficient α is also perfectly consistent with the literature [2] and the value of 3% that we used in section S4 to estimate the exciton density. We show in Supplementary Figure S11, the influence of both α and τ_0 on the results of the trimolecular model. It shows that α and τ_0 are linked (a larger α requires a smaller τ_0 to give the same results).

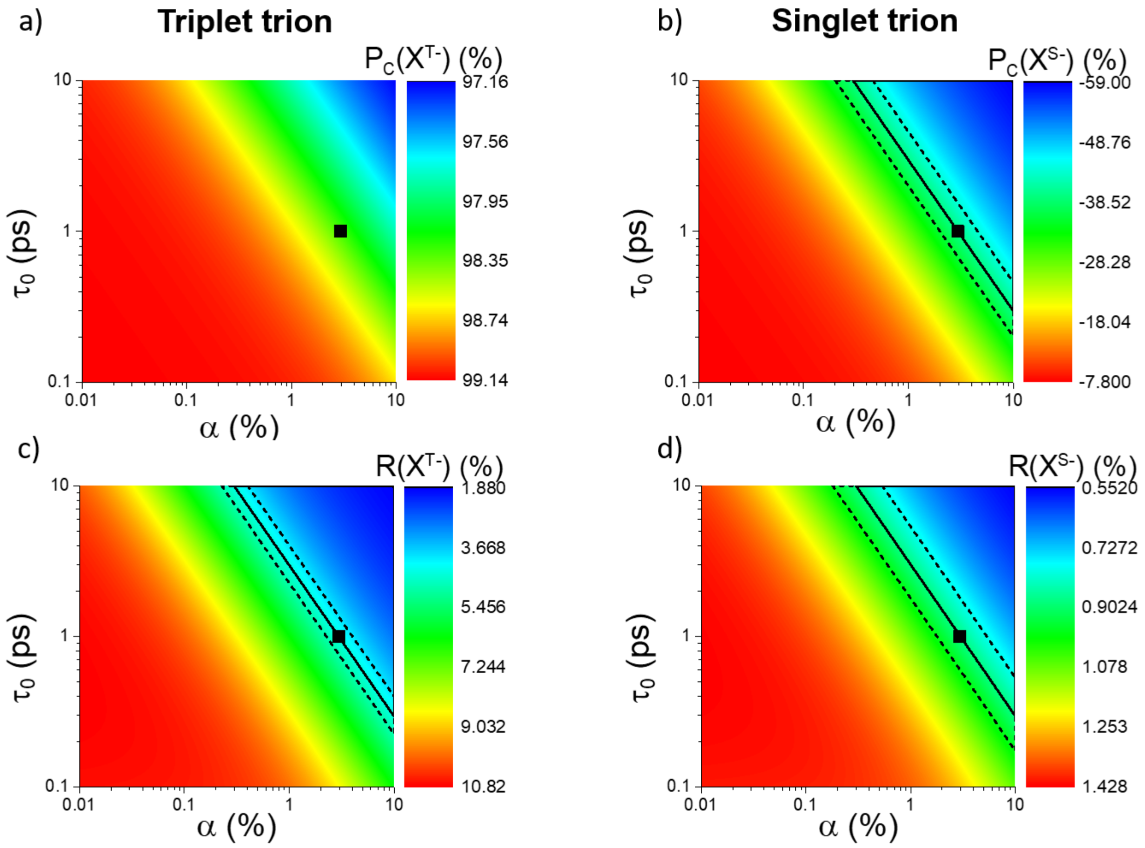

*Supplementary Figure S11: **Trimolecular model**: Influence of the absorption coefficient α and the bright exciton lifetime τ_0 on the calculated degrees of circular polarization for (a) triplet and (b) singlet trions and on the ratio R (c) and (d). The black data point correspond to the result of the fit . The black solid lines are the contour plot of the experimental data ($P_c(X^{T-})$=91%, $P_c(X^{S-})$=-40%, $R(X^{T-})$=4.4 and $R(X^{S-})$=0.88) and the dashed lines are the contour plots of the experimental data with a tolerance of ±10%. All other parameters are fixed to the values written in the text.*

Finally, we discuss about the trimolecular formation coefficients. We show in Figure S12, the influence of C_B and C_D on $P_c(X^{T-})$, $P_c(X^{S-})$, $R(X^{T-})$ and $R(X^{S-})$. We clearly see that for C_D>10^{-21} cm^{-4}.ps^{-1}, the results are only sensitive to the ratio C_B/C_D which in our fit is close to 1000. Interestingly, this ratio also fix the ratio between the PL intensity of bright and dark trions. In Figure 2b of the main text we observed that X^{T-}, X^{S-} and X^{D-} have a similar intensity for linear excitation. With our model, we find that X^{D-} is 10 times larger than $X^{T-}$and X^{S-} which is not so far from our experimental observations (given that the light emitted by X^{D-} is not well collected due to its in-plane direction). We would also like to mention that with our model, the total PL intensity (bright trions + dark trions) is the same for circular and linear excitation.

In conclusion, despite its simplicity, our model describes rather well our experimental observations with only a few set of parameters with values perfectly consistent with their scientific meaning.

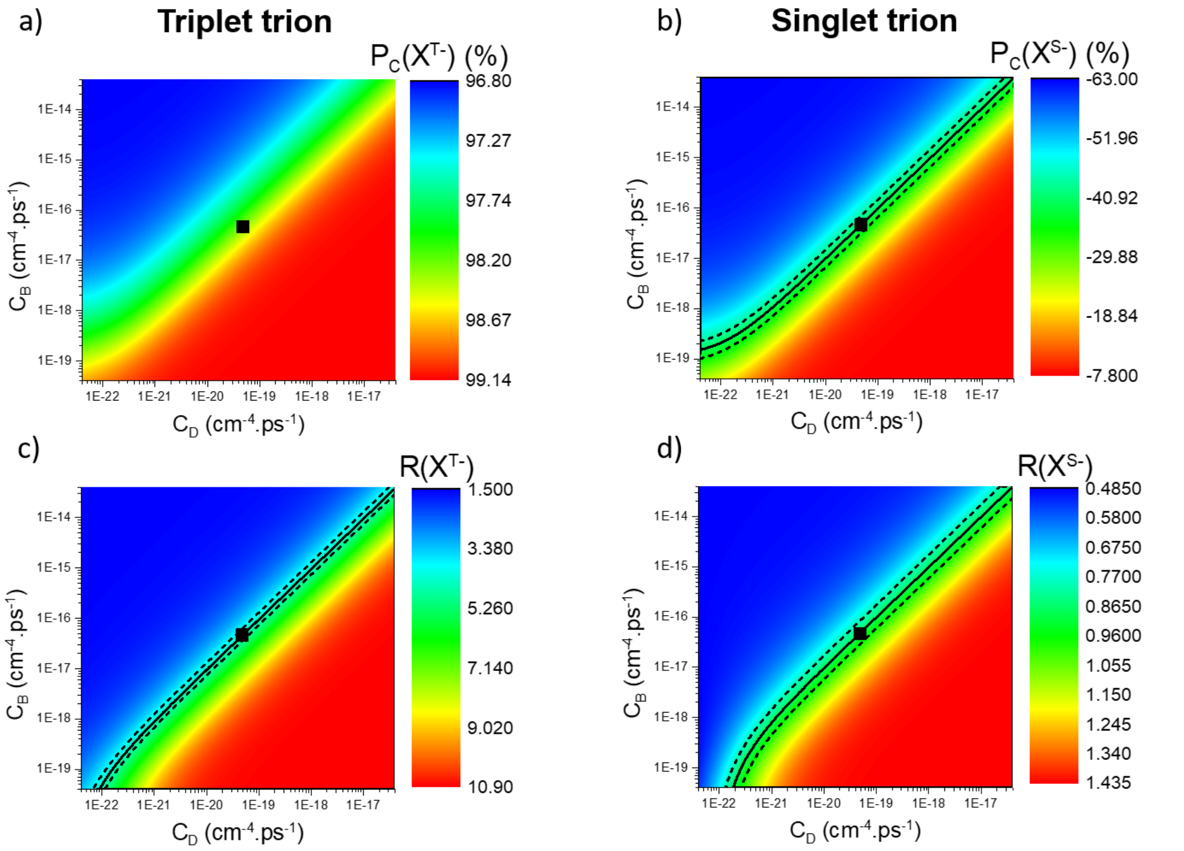

*Figure S12: **Trimolecular model**: Influence of the trimolecular formation coefficients of the bright and dark trions C_B and C_D on the calculated degrees of circular polarization for (a) triplet and (b) singlet trions and on the ratio R (c) and (d). The black data point correspond to the result of the fit . The black solid lines are the contour plot of the experimental data ($P_c(X^{T-})$=91%, $P_c(X^{S-})$=-40%, $R(X^{T-})$=4.4 and $R(X^{S-})$=0.88) and the dashed lines are the contour plots of the experimental data with a tolerance of ±10%. All other parameters are fixed to the values written in the text.*

Supplementary Note 11. Power dependence in WS_2

We present the circular polarization degree P_c and the ratio R for both triplet and singlet trions in WS_2 ML as function of laser power. The results are very similar to WSe_2 (Figure 2e and f of the main text): when the excitation power decreases, $P_c(X^{T-})$and $P_c(X^{S-})$ converge to the same value (around 30% for WS_2) and $R(X^{T-})$ and $R(X^{S-})$ converge to 1.

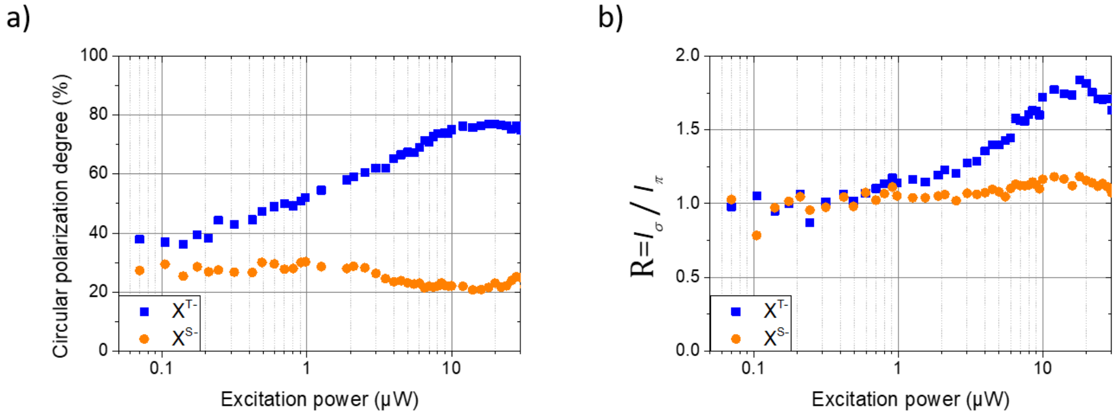

Supplementary Figure S13: (a) Circular polarization degree at the peak of triplet and singlet as a function of excitation power. (b) Ratio of PL intensities between circular and linear excitations at the peak for both triplet and singlet as a function of excitation power.

References

[1] C. Robert, H. Dery, L. Ren, D. Van Tuan, E. Courtade, M. Yang, B. Urbaszek, D. Lagarde, K. Watanabe, T. Taniguchi, T. Amand, and X. Marie, *Measurement of Conduction and Valence Bands g-Factors in a Transition Metal Dichalcogenide Monolayer*, Phys. Rev. Lett. **126**, 067403 (2021).

[2] Y. Li, A. Chernikov, X. Zhang, A. Rigosi, H. M. Hill, A. M. van der Zande, D. A. Chenet, E.-M. Shih, J. Hone, and T. F. Heinz, *Measurement of the Optical Dielectric Function of Monolayer Transition-Metal Dichalcogenides: MoS2, MoS2, WS2, and WSe2*, Phys. Rev. B **90**, 205422 (2014).

[3] J. Zipfel, K. Wagner, J. D. Ziegler, T. Taniguchi, K. Watanabe, M. A. Semina, and A. Chernikov, *Light–matter Coupling and Non-Equilibrium Dynamics of Exchange-Split Trions in Monolayer WS2*, J. Chem. Phys. **153**, 034706 (2020).

[4] E. Liu, J. van Baren, T. Taniguchi, K. Watanabe, Y.-C. Chang, and C. H. Lui, *Landau-Quantized Excitonic Absorption and Luminescence in a Monolayer Valley Semiconductor* Phys. Rev. Lett. **124**, 097401 (2020).
